# Oat Plant Amyloids for Sustainable Functional Materials

**DOI:** 10.1002/advs.202104445

**Published:** 2021-12-20

**Authors:** Jiangtao Zhou, Ting Li, Mohammad Peydayesh, Mattia Usuelli, Viviane Lutz‐Bueno, Jie Teng, Li Wang, Raffaele Mezzenga

**Affiliations:** ^1^ Department of Health Sciences and Technology ETH Zurich Zurich 8092 Switzerland; ^2^ School of Food Science and Technology National Engineering Laboratory for Cereal Fermentation Technology Jiangnan University Lihu Road 1800 Wuxi 214122 China; ^3^ Department of Materials ETH Zurich Zurich 8093 Switzerland

**Keywords:** amyloid fibrils, functional amyloid materials, plant protein, reversible amyloid, sustainability

## Abstract

Amyloid functional materials from amyloid fibril building blocks, produced in vitro from amyloidogenic natural proteins or synthetic peptides, show diverse functionalities ranging from environmental science and biomedicine, to nanotechnology and biomaterials. However, sustainable and affordable sources of amyloidogenic proteins remain the bottleneck for large‐scale applications, and to date, interest remains essentially limited to fundamental studies. Plant‐derived proteins would be an ideal source due to their natural abundance and low environmental impact. Hereby oat globulin, the primary protein of oat plant (*Avena sativa*), is utilized to yield high‐quality amyloid fibrils and functional materials based thereof. These fibrils show a rich multistranded ribbon‐like polymorphism and a fibrillization process with both irreversible and reversible pathways. The authors furthermore fabricate oat‐amyloid aerogels, films, and membranes for possible use in water purification, sensors, and patterned electrodes. The sustainability footprint of oat‐amyloids against other protein sources is demonstrated, anticipating an environmentally‐efficient platform for advanced materials and technologies.

## Graphical abstract

Plant‐derived protein is an ideal source to scale‐up amyloid‐material applications due to their natural abundance and low environmental impact. Hereby, the authors found oat globulin could yield high‐quality amyloid fibrils, showing multistranded ribbon‐like polymorphism and both irreversible and reversible fibrillization pathways. The authors anticipate these sustainable oat‐amyloids would be an environmentally‐efficient platform for advanced materials.

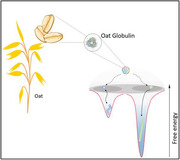

## 1 Introduction

Amyloids are filamentous structures self‐assembled from amyloidogenic proteins or polypeptides.^[^ ^1^ ^]^ In addition to functional and pathological amyloids in vivo,^[^ ^2^ ^]^ the synthesis of artificial amyloid fibrils in vitro is a promising route for designing biomaterials.^[^ ^1b,d^ ^]^ Recently, advances in artificial functional amyloid‐based biomaterials have soared due to their outstanding biocompatibility, biodegradability, and high mechanical and physiochemical performances.^[^ ^1^, ^3^ ^]^ These features have favored the use of amyloid fibrils for artificial functional materials in many biomedical and biomaterial applications,^[^ ^1^, ^4^ ^]^ such as drug/nutrition delivery,^[^ ^5^ ^]^ antitumor therapy,^[^ ^6^ ^]^ biomimetic composites,^[^ ^7^ ^]^ and water purification.^[^ ^8^ ^]^

Unfortunately, current amyloid‐based biomaterials are nearly exclusively derived from animal‐based proteins,^[^ ^9^ ^]^ synthetic chemistry,^[^ ^3^ ^]^ or synthetic biological systems.^[^ ^10^ ^]^ Sustainability and affordability are the major barriers for manufacturing amyloid biomaterials and applying them into real‐scale applications, so that seeking an environmentally‐friendly source of amyloid remains a critical bottleneck. Plant‐derived proteins could be an ideal source to address this issue, and increasing efforts are being made to seek relevant plant amyloidogenic protein sources,^[^ ^11^ ^]^ such as rice, soy, pea, and potato. Yet, the translation into functional materials and technologies remains to be demonstrated, as for their scalable production. The reason for these pitfalls is threefold: most plants typically have a highly diverse protein composition that complicates the purification process; protein allergenicity in some plant sources, such as soy and pea,^[^ ^12^ ^]^ can be an issue for biological applications; identifying amyloidogenic protein from numerous candidates in plant remains elusive.

**Figure 1:**
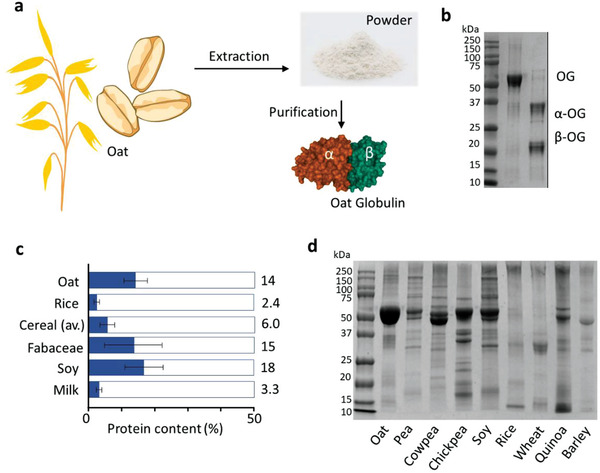
Oat globulin extraction process and comparison with proteins from other plant sources. a) Schematic of extraction and purification of oat globulin from oat grain. b) SDS‐PAGE of oat globulin and reduced oat globulin. Oat globulin in the presence of 1% SDS, 31.3 mm Tris‐HCl, 12.5% glycerol (oat globulin), or of 1% SDS, 2.5% *β*‐mercaptoethanol, 31.3 mm Tris‐HCl, 12.5% glycerol (reduced oat globulin) were tested, respectively. c) Protein content from different sources including oat, rice, cereal (average), fabaceae, soy, and milk. Oat has a protein content of 14%, the highest in all cereal grains^[^ ^18^ ^]^ and comparable to bean and soy. d) Protein diversity of oat, pea, cowpea, chickpea, soy, rice, wheat, quinoa, and barley. Oat globulin is the dominating fraction in oat protein, while other plants have a broader diversity in their protein composition.

In this work, we present a sustainable and non‐allergenic plant protein fraction, oat globulin (OG), as a source for high‐quality amyloid fibrils and functional materials based thereof. Oat plant protein as a possible source for amyloid fibrils has been suggested before by us^[^ ^13^ ^]^ and other groups;^[^ ^14^ ^]^ however, it is clear from the extremely scarce literature available that without a proper purification and fibrillization protocol, the extraction yield is insufficient and the polymorphism of the amyloids remains totally hidden.^[^ ^14b^ ^]^ A schematic of protein extraction from oat grain and further purification of the OG fraction is shown in **Figure** **1**a. Monomeric OG has a molecular weight of about 55 kDa and consists of two subunits^[^ ^15^ ^]^ (*α* and *β*, with molecular weights of 32 and 22 kDa, respectively) linked by disulfide bridges (Figure 1b; Figure S1, Supporting Information). Oat plant, a unique cereal from the grass family, has a protein content as high as 11–17% (Figure 1c). Such a value is considerably larger than the one of other cereal plants^[^ ^16^ ^]^ (average of 6%) and rice, a broadly‐recognized pillar in global nutrition, which accounts for only 2.4% protein content. The legume family, to which pea and soy belong, exhibits a slightly higher protein proportion over oat; however, their protein composition is highly diverse (Figure 1d), with consequent challenges in protein purification and protein yield rate.^[^ ^11^, ^14^, ^17^ ^]^ Moreover, legume protein sources, such as soy and pea,^[^ ^12^ ^]^ may provoke allergic reactions that hamper biomedical and food applications such as nutrition delivery.^[^ ^5a^ ^]^ By contrast, OG is the predominant protein fraction in oat storage protein,^[^ ^16^ ^]^ accounting for up to 80% of entire oat proteins (Figure 1d; Figure S2, Supporting Information). This feature is a key advantage to ensure a high yield of OG and consequently its amyloid fibrils.

## 2 Results and Discussion

### 2.1 Oat Globulin Amyloid Fibril Formation and Analysis

**Figure 2:**
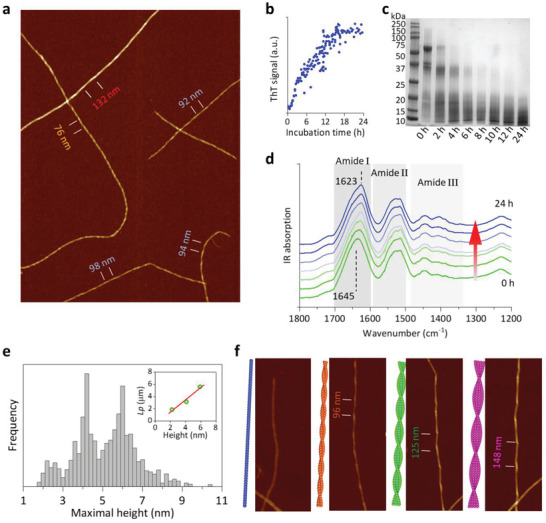
Characterization and analysis on the oat globulin mature amyloid fibril. a) AFM height image of oat globulin amyloid fibrils. Hierarchical fibrils show a clear periodic height fluctuation with different periodicities. b) ThT fluorescence assay of oat globulin formation. ThT signal immediately increased after incubation at 90 °C and gradually reached a plateau after 18 h. c) SDS‐PAGE pattern of protein solution during incubation, indicating a gradual hydrolysis of monomeric oat globulin. d) Evolution of FTIR spectra showing the transition from helical and random coil structures (1645 cm^−1^) to *β*‐sheets (1623 cm^−1^). e) Distribution of oat globulin fibril maximum heights, showing three main peaks, 2.2, 4.1, and 6.0 nm, that are related to different families of fibrils. Inset shows the linear correlation between persistence length (*L*_p_) and fibril height. f) Proposed schemes and corresponding AFM images of multistranded left‐handed twisted ribbon fibrils with one, two, three, and four protofilaments.

To this end, we developed a comprehensive protein purification and fibrillization process to produce OG and favor its self‐assembling into amyloid fibrils (see Experimental Section). The purified OG was incubated at high temperature (90 °C) and low pH: heat‐denaturation and hydrolysis led to the formation of mature rigid amyloid fibrils, as shown in atomic force microscope (AFM) and transmission electron microscope (TEM) images (**Figure** **2**a; Figure S3, Supporting Information). These fibrils could reach several micrometers in length with a clear periodicity arising from their self‐twisting behavior.^[^ ^9^, ^19^ ^]^ The signal from the thioflavin T (ThT) fluorescence assay (Figure 2b) reached a plateau after 16 h of incubation. During fibrillization at pH 2, the positively charged OG (Figure S4, Supporting Information) was gradually hydrolyzed into polypeptides (Figure 2c) that re‐assembled into amyloid fibrils. The *β*‐strand structure was confirmed by attenuated total reflectance Fourier‐transform infrared spectroscopy (ATR‐FTIR, Figure 2d). The Amide I band (1700–1600 cm^−1^), assigned to the vibrational absorption of C═O stretching, is highlighted on the IR spectrum. The absorption peak in Amide I shifted from 1645 cm^−1^ to a lower wavenumber (1623 cm^−1^), pointing out the secondary structural transition from random coil polypeptides to *β*‐sheet rich amyloid fibrils in solution.

We then performed a statistical polymer physics analysis of individual amyloid fibrils from AFM images to understand the underlying fibrillization mechanism.^[^ ^9a^ ^]^ To avoid the artifacts from AFM tip convolution effect, we analyze the maximum of each cross section along the ridge of each fibril contour.^[^ ^9^, ^20^ ^]^ The maximum height distribution of amyloid fibrils is presented in Figure 2e, with three main populations with heights ranging from 1.5 to 11 nm. The three dominating groups are centered at 2.2 ± 0.5, 4.1 ± 0.5, and 6.0 ± 0.7 nm, suggesting a hierarchical height distribution associated to multistranded amyloid fibrils. Similar to milk‐derived *β*‐lactoglobulin (*β*‐lg) amyloid fibrils,^[^ ^9a^ ^]^ this general model involves individual protofilaments which are laterally packed into different families of twisted ribbon multistranded fibrils.^[^ ^9a^ ^]^ In Figure 2f, the schematic representations of different families of multistranded twisted ribbon‐like fibrils are presented, with AFM image illustrations in each corresponding family. The single protofilament with no periodic height fluctuation and a diameter of 2.2 nm (Figure 2e) is the building block evolving into higher‐ordered multistranded fibrils. The double‐ and triple‐stranded twisted ribbon fibrils, peaked at 4.1 and 6.0 nm, respectively, are the most common populations among the OG rigid amyloid fibrils (Figure 2e). The thickest fibril with 8.4 nm in height was identified as a four fold‐stranded amyloid fibril. The hierarchical heights of traced fibrils agree with the multistranded packing scheme (Figure 2e) confirming a multistranded twisted ribbon polymorphism. A deeper mesoscopic characterization (Figure 2a,f) shows that OG fibrils are left‐handed under equilibrium conditions. Although the periodicity increases linearly with maximum height (Figure 2f), snapshots in Figure S5 show that this pitch may vary, especially while fibrils are forming. This arises as the twisting of amyloid fibrils is a dynamic equilibrium of different contributions arising from intrinsic chirality, electrostatic interactions, and mechanical strain.^[^ ^21^ ^]^ Further examples of OG fibril structural polymorphs are presented in Figures S5 and S6.

As a further support to the proposed multistranded twisted ribbon model, the persistence length of each family of rigid fibrils, extracted by the 2D worm‐like semiflexible polymer model (see Figure S7, Supporting Information) was found to follow a linear relationship with the number of filaments in a mature fibril (Figure 2e inset; Figure S7, Supporting Information). For the proposed ribbon‐like polymorphism, the scaling behavior of the persistence length is indeed expected to be $l_{p}\approx \;nr_{0}^{4}E/k_{B}T$,^[^ ^9a^ ^]^ where *n* is the number of protofilaments, *r*_0_ is the radius of a single filament, *E* is the Young's modulus of the polymer, *k*_B_ is the Boltzmann constant, and *T* is the absolute temperature; this scaling law is in perfect agreement with our experimental data.

### 2.2 Coexistence of Oat Globulin Mature Rigid fibril and Worm‐Like Fibril

**Figure 3:**
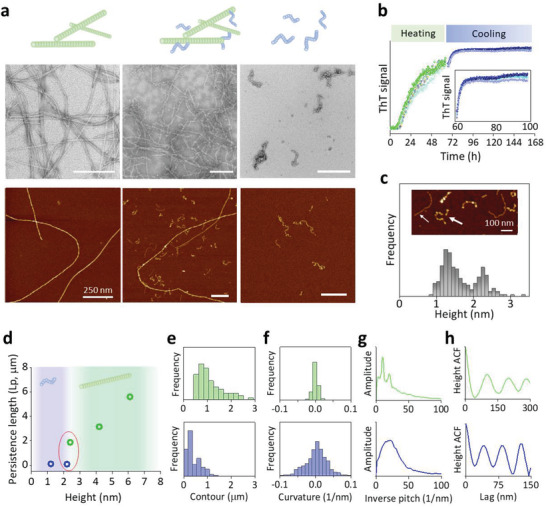
Formation and coexistence of mature fibrils and flexible worm‐like fibrils. a) TEM and AFM images of the mature fibril, coexistence of mature and worm‐like fibril, and elongated worm‐like fibrils. b) ThT assay of oat globulin solution during a heating and following cooling treatment corresponding to mature (green) and worm‐like (blue) fibrillization, respectively. To automatically sampling ThT signal, the protein solution was first incubated at 65 °C in the reader; ThT signal reached the equilibrium phase after 60 h. Then, the following incubation at room temperature enabled a further aggregation of worm‐like fibrils, with detailed curves in the inset. c) Histogram of worm‐like fibril maximum height, that shows two families of worm‐like fibrils with different heights as illustrated in the inset. d) Persistence length of each analyzed family of worm‐like fibrils (blue) and mature rigid fibrils (green). The worm‐like fibrils, irrespectively of the family they belong to, have a persistence length of ≈100 nm. Diversely, the persistence length of rigid fibrils is a function of the family they belong to, and can be as high as several µm. Families of worm‐like and rigid fibrils that have a similar average height, show instead a difference of one order of magnitude between their persistence lengths (as highlighted by the red circle). Comparison of molecular and morphological features between mature fibril and worm‐like fibril, including e) contour length, f) curvature histograms, g) periodicity analysis and h) pitch estimation.

Interestingly, the polymorphism of oat amyloid fibrils can be enriched beyond the rigid amyloid fibrils obtained by heat‐denaturation at 90 °C (**Figure** **3**a, left panel). After cooling‐incubation at room temperature (RT) for a few days, short worm‐like fibrils started to emerge and coexisted with mature rigid fibrils (Figure 3a, middle panel). These short and flexible worm‐shaped fibrils could be detected after 2 days of incubation at RT and proliferated rapidly. To gain insight into their formation kinetics, we followed their evolution through a ThT assay (Figure 3b): after the first ThT plateau under heat‐treatment, another ThT signal increase was detectable in the following cooling‐process, revealing further *β*‐aggregation in concomitance with worm‐like fibril formation. These short fibrils were then isolated and after a long‐time (50 days) incubation, elongated to up to 500 nm in length, yet maintaining a highly flexible shape (Figure 3a right panel; Figure S8, Supporting Information). A structural investigation by FTIR and circular dichroism (CD) spectrometer (Figures S9 and S10) showed that both mature rigid fibril and worm‐like fibril are characterized by intermolecular *β*‐sheet secondary structure.

A further morphological characterization (Figure 3c) revealed two families of worm‐like fibrils, with maximum height peaked at 1.2 ± 0.3 and 2.3 ± 0.2 nm, representing single‐stranded and twisted fibrils, respectively. The persistence length of both families of worm‐like fibrils is around 100 nm (Figure 3d; Figure S8, Supporting Information), ten times smaller than that of mature rigid fibrils. The different order of magnitude in persistence length holds true even when considering two families of rigid and flexible amyloids with comparable heights (2.2 and 2.3 nm, respectively). Their difference, as highlighted in Figure 3d with a red circle, infers that the worm‐like fibrils must have one order of magnitude lower Young's modulus, that is, highly flexible amyloid cores. This might be associated with a less‐rigid *β*‐strand backbone or a weak LARKS structural motif,^[^ ^22^ ^]^ whereas mature rigid fibrils usually contain robust fibril's cores or structural motifs, such as steric zipper.^[^ ^23^ ^]^ A more comprehensive comparison of morphological features at the molecular level (Figure 3e–h), including contour length, curvature, periodicity, and pitch size analysis, reveals that OG mature fibrils are long and rigid assemblies with a twisted periodicity, while worm‐like fibrils are short and highly flexible.

### 2.3 Irreversibility and Reversibility of Oat Globulin Amyloid Fibril

**Figure 4:**
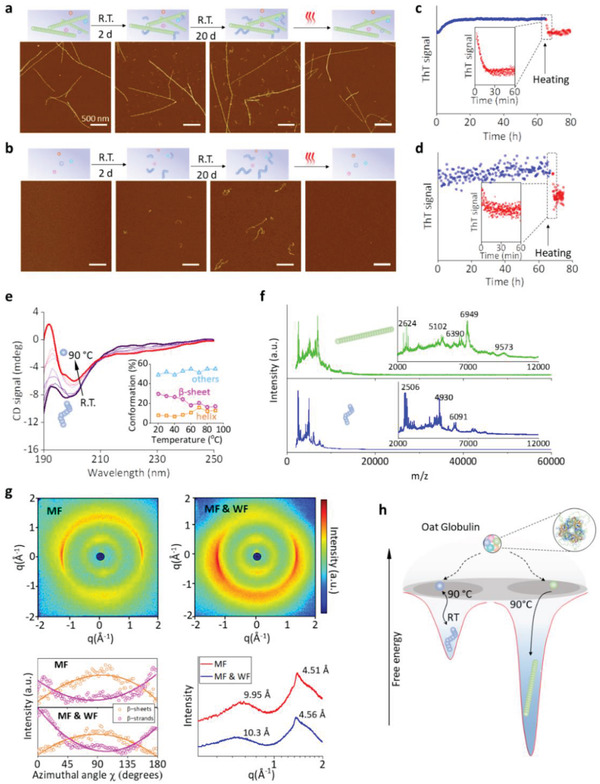
The reversibility of worm‐like fibrils and oat globulin fibrillization mechanism. Forming and dissembling of worm‐like fibrils over the cooling and heating processes respectively in the a) presence and b) absence of mature fibrils. ThT signal during the cooling (blue) and heating (red) incubation processes in the c) presence and d) absence of mature fibrils. The insets show the details in a higher time resolution. e) CD spectra of worm‐like fibril solutions overheating from 20 to 90 °C. Inset shows secondary structural variation obtained from the analysis of the collected CD spectra. f) MALDI‐MS spectra of mature rigid and worm‐like fibrils. g) WAXS 2D‐scattering patterns, azimuthal and radial WAXS profile of the film of mature oat fibrils (MF), and the hybrid of mature and worm‐like fibrils (MF and WF). h) Energy landscape of oat globulin mature rigid fibrils and worm‐like fibrils.

In line with the above picture, we discovered thermal reversibility in the case of flexible worm‐like fibrils. **Figure** **4**a shows the formation of worm‐like fibrils in the presence of mature fibrils: a freshly heated protein solution with pure mature rigid fibrils (left) exhibited a large amount of short worm‐like fibrils after 2‐days incubation at RT (middle). These worm‐like fibrils further elongated, reaching lengths up to hundreds of nanometers after 20 days, yet they disappeared immediately upon further heating (right). This trend was verified by ThT assay (Figure 4c): ThT signal increased steadily at RT while worm‐like fibrils were forming, but dropped within 15 min after sudden heating, indicating the melting of these worm‐like fibrils. To confirm this behavior, we repeated this experiment in the absence of mature fibril (Figure 4b): the filtrate of freshly heated protein solution was collected (left) and incubated at RT; worm‐like fibrils were detected after 2 days (middle), which further elongated to up to 500 nm after 20 days and dissembled immediately after heating‐treatment (right), as again confirmed by ThT assay (Figure 4d). We then traced, using CD spectroscopy, the evolution of *β*‐sheet content in the probed protein solution, over the heat‐induced disassembly of the worm‐like fibrils (Figure 4e). As the temperature rose and the worm‐like fibrils disassembled, the spectrum varied continuously and systematically. Secondary structural analysis by Bestsel fitting^[^ ^24^ ^]^ suggested a drop of *β*‐sheet content accompanied by an increase of helical and other secondary structures, including random coil and loops.

To gain a molecular insight of the chemical nature of both mature and worm‐like fibrils, matrix‐assisted laser desorption/ionization mass spectrometry (MALDI‐MS) was performed. In Figure 4f, mature fibrils are characterized by a dominating peak (6949), while worm‐like fibrils presented clearly shorter polypeptides with two main peaks (2506 and 4930). This indicates the important fact that mature rigid fibrils and worm‐like fibrils are originated from different polypeptides hydrolyzed from monomeric OG, with an estimated yield of amyloid fibril of around 40% (Figure S11, Supporting Information). We further characterized the atomistic structural properties of the formed fibrils through wide angle X‐ray scattering (WAXS), which gives insights into the amyloid structural organization at the molecular level. The incident X‐ray beam was shot on an oat amyloid film, parallel to its surface,^[^ ^25^ ^]^ and gave rise to typical cross‐*β* scattering patterns, as the hallmark of amyloids, with 90°‐shifted strong azimuthal intensity peaks in both the rigid fibril film and rigid‐flexible fibril composite film (Figure 4g). The scattering intensity that reaches its maximum at the equatorial reflections is associated with hydrogen‐bonded inter‐*β*‐strands, while the one reaching maxima at the meridians is associated with the *β*‐sheet stacking distances.^[^ ^26^ ^]^ These structural features are found to be respectively 4.51 and 9.95 Å for mature rigid fibrils, and 4.56 and 10.3 Å for the composite film, in agreement with structural diffraction patterns of amyloids.

Figure 4h summarizes the origin and fibrillization mechanism of OG irreversible mature and reversible worm‐like fibrils. From a protein energy landscape perspective, amyloid is the most stable thermodynamic state;^[^ ^27^ ^]^ however, certain amyloid polymorphs are metastable and occupy therefore relative minima.^[^ ^19^, ^28^ ^]^ Here, monomeric OG first hydrolyzed during heat‐denaturation. It is plausible to assume that the worm‐like amyloid fibrils formed by shorter peptides have a lower energy gain from the assembly of short peptide fragments, leading to metastable aggregates, whereas this energy gain may be larger for longer peptides forming irreversible amyloids, positioning them lower in the energy landscape. This picture is consistent with the persistence length analysis we performed (Figure 3d), which pointed out the intrinsic softer nature of worm‐like fibrils compared to rigid amyloids, and hence, a lower density of the H‐bonding network.

### 2.4 Biomaterial Applications of Oat Globulin Amyloid Fibrils

**Figure 5:**
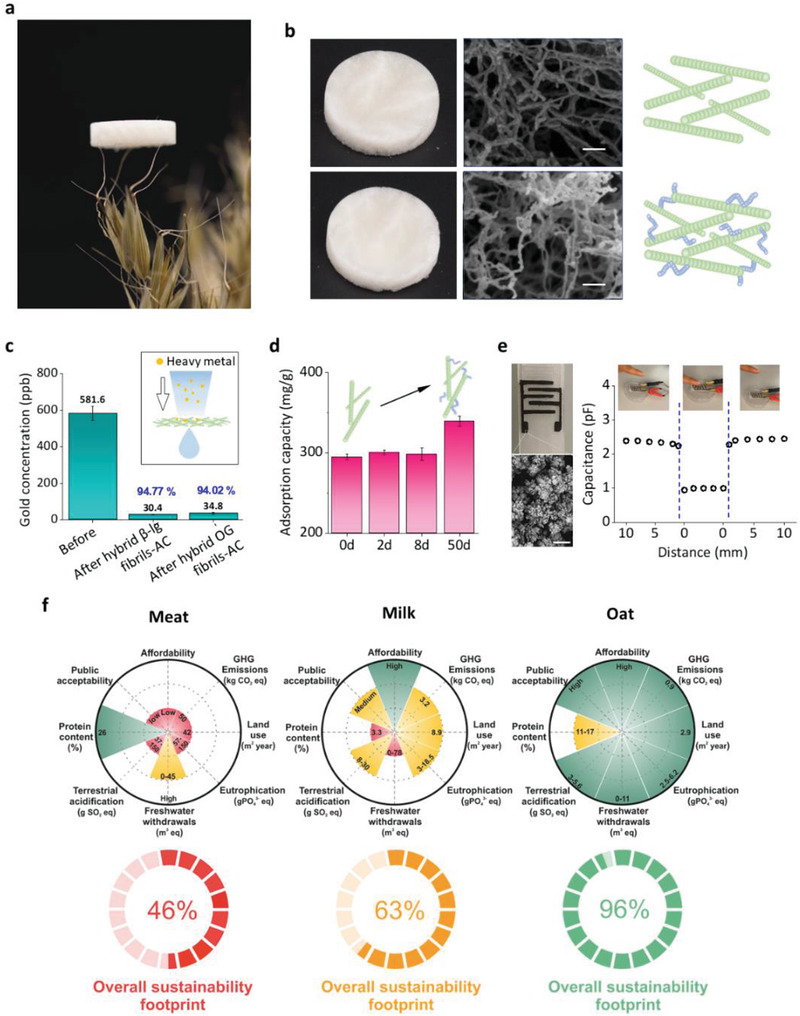
Functional material applications and sustainability of oat amyloid fibrils. a) Oat amyloid based aerogel on the top of the awns of an oat spikelet. b) Photo (left), SEM images (middle), and schematics (right) of freeze‐dried aerogel from oat globulin mature fibril (top) and hybrid of mature and worm‐like fibrils. Scale bar: 100 nm. c) Water purification performance of *β*‐lactoglobulin/oat globulin fibrils‐active carbon hybrid membranes, with purification efficiency indicated (blue). d) Heavy metal adsorption capacity of oat mature fibril and hybrid of mature fibrils and different amounts of worm‐like fibrils incubated from 2 to 50 days. e) Au‐amyloids interdigital electrode and its capacitance variation with a finger approaching to and retracting from the electrode. Scale bar = 500 nm. f) Sustainability footprint of meat protein, milk protein, and oat protein with multiple factors considered, including affordability, GFG emissions, land use, eutrophication, freshwater withdrawals, terrestrial acidification, protein content, and public acceptability. Scores in each discriminant are shown by red (low), yellow (medium), and green (high performance). The overall sustainability footprint indicates that oat is the most sustainable source of protein in this comparison.

Having established the molecular assembly of reversible and irreversible oat plant amyloids, we next demonstrate their use in functional materials and technologies and their superior sustainability over alternative animal protein sources. We fabricated an OG amyloid‐based aerogel and, as illustrated in **Figure** **5**a, placed it on the top of the awns of an oat spikelet to highlight its lightness. In Figure 5b, freeze‐dried aerogels made by pure mature amyloid fibrils (top) and hybrid mature and worm‐like fibrils (bottom) are shown. Scanning electron microscopy (SEM) images confirm that mature‐fibril aerogels are composed of thick rigid fibrils, while the hybrid aerogel contains both flexible worm‐like fibrils and mature fibrils. In Figure S12, Supporting Information, we also show the possibility of synthesizing ion‐induced hydrogel based on OG mature amyloids.

We then studied the performance of oat amyloid‐based membranes for state‐of‐the‐art water purification, fabricated according to the previously published procedures.^[^ ^8b^ ^]^ The results (Figure 5c) demonstrate that this oat‐based amyloid membrane features similar high‐performance in heavy metal (Au as a model pollutant) removal compared to the most frequently used membranes constituted by milk‐based *β*‐lg amyloid fibrils. Further test with a higher concentration of gold (Figure S13, Supporting Information) indicates that these two classes of amyloids exhibit a comparable adsorption capacity for heavy metal removal. In addition, the adsorption capacity of pure oat mature fibrils can be increased significantly by different amounts of coexisting worm‐like fibrils (Figure 5d). This might be due to either an increased surface area in the presence of the worm‐like fibrils, or additional protein‐binding domains to absorb heavy metals^[^ ^16^ ^]^ featured by the different peptide composition of worm‐like amyloid fibrils.

As a further demonstration of the potential of oat amyloids in nanotechnology, protein‐based Au‐nanoparticle (NP) coated interdigital electrodes were produced (Figure 5e), which could find application in biocompatible and biodegradable electronics and soft robotics.^[^ ^29^ ^]^ The capacitance of this electrode was monitored while putting a finger in contact with it. The capacitance decreased while the finger was approaching, dropped while touching, and restored upon finger removal. Moreover, we proved that the capacitance in this AuNP‐amyloid electrode is pressure‐dependent (Figure S14, Supporting Information), showing the potential of using it as a high‐sensitivity pressure‐electric sensor. Additionally, this protein‐based hybrid material was shown to feature a good electrical conductivity (Figure S15, Supporting Information).

The real advantage of translating from animal‐based to plant‐based amyloids becomes evident in Figure 5f, where we evaluate the sustainability footprint of oat production compared to meat and milk, as benchmark protein sources. To include all three pillars of sustainability, that is, techno‐economic, environmental, and social, eight factors including protein content, public acceptability, affordability, green‐house gas emissions, land use, eutrophication, freshwater withdrawals, and terrestrial acidification were considered. The environmental data were adapted from the study by Poore and Nemecek,^[^ ^30^ ^]^ and the performance of each protein source in each factor was ranked as low (*i = 1*), medium (*i = 2*), and high (*i = 3*) level score.^[^ ^8a^ ^]^ As observed, almost all factors score better in the case of oat over the other two sources. An overall sustainability footprint was calculated by summing up the individual components as $100\%\cdot \sum_{j=1}^{8}{\left(\frac{i}{3}\right)}_{j}\cdot \frac{1}{8}$, where each of the *j* = 8 discriminants carries a weight between 1/3 and 1 depending on the score *i*.^[^ ^31^ ^]^ The overall sustainability footprint for meat, milk, and oat was around 46%, 63%, and 96%, respectively, demonstrating oat as the most sustainable source of protein among the considered cases.

## 3 Conclusion

In summary, we have shown that OG, a non‐allergic and primary protein from the protein‐rich oat plant, can be used as a sustainable source for producing high‐quality amyloid fibrils and functional materials based thereof. The mesoscopic polymorphism of these amyloids can be enriched by thermal processing, leading to the formation of either rigid irreversible amyloids or semiflexible, reversible amyloid fibrils, which can, in turn, be used for the formation of aerogels, water membranes, electrodes, or sensors. The superior sustainability fingerprint of oat amyloids compared to other sources of animal proteins make them an ideal candidate for scaling up the use of artificial amyloids in real‐scale materials and technologies.

## 4 Experimental Section

### Protein Purification

OG was extracted from oat flour. First, oat seeds were milled to powder using a grinder. The oat flour was defatted by petroleum ether for 12 h with a ratio of 1:10 in a fume hood. Subsequently, the suspension was centrifuged at 5000 g for 15 min. The defatted procedure was replicated three times. The resulting precipitates were gathered and dried in a fume hood overnight. The defatted oat flour was dispersed in distilled water (1:10) and then the dispersion was adjusted to pH 10 to extract oat protein isolates after 2 h. Then the dispersion was centrifuged at 5000 g for 15 min to obtain the oat protein solution. The oat protein solution was adjusted to pH 7, then centrifuged again at 5000 g for 15 min to remove water‐soluble albumin. Subsequently, the resulting protein precipitates were dispersed in a 5% NaCl solution and stirred for 2 h. The solution was centrifuged at 5000 g for 15 min to collect the supernatant, the pH of which was adjusted to 4.5 and centrifuged for 15 min at 5000 g. The resulting OG precipitates were re‐adjusted to pH 7 and dialyzed for 24 h using 3.5 kDa membranes. Finally, the OG powder was obtained by a freeze‐drying process.

### Irreversible and Reversible Amyloid Fibril Preparation

Freeze‐dried OG powder was first dissolved in Milli‐Q water (pH 2) at a concentration of 2 wt%. After stirring 5 min in ambient condition, the solution was then re‐adjusted to pH 2. Then, the dissolved protein solution was incubated with a heat treatment (90 °C) by means of an oil bath for 18 h while stirring at 350 rpm, to yield the irreversible OG mature rigid amyloid fibrils. To obtain hybrid irreversible and reversible amyloid solution, the heat‐treated solution was further incubated in a cooling process at RT (≈25 °C) for 2–50 days. To obtain the pure reversible fibrils, the first‐step heat‐treated solution was filtered with a MWCO 100 kDa filter at 4 °C. The filtrate solution was then incubated at RT under the ambient condition for 2–50 days.

### Atomic Force Microscope Imaging and Statistical Analysis

Aliquots of protein solutions were collected and diluted to 0.02 wt% with Milli‐Q water (pH 2). Immediately, an aliquot (10 mL) of diluted protein solution was deposited on a freshly cleaved mica, incubated for 2 min, rinsed with Milli‐Q water and dried by a gentle flow of nitrogen gas. AFM measurements were carried out by a Bruker multimode 8 scanning probe microscope (Bruker, USA) with an acoustic hood to minimize vibrational noise. AFM imaging was operated in soft tapping mode under the ambient condition, using a commercial silicon nitride cantilever (Bruker, USA) at a vibration frequency of 150 kHz, and a relatively soft tip‐sample interaction was applied. AFM images were flattened using Nanoscope 8.1 software (Bruker, USA), and no further image processing was applied. The statistical analysis of flattened AFM images was carried out using the FiberApp software.^[^ ^20^ ^]^ The amyloid fibrils were traced by selecting the maximal height in every cross section along the contour of each individual amyloid fibril.^[^ ^32^ ^]^ An additional custom‐made script was applied to calculate the maximal height of the twisted fibrils and to group the traced fibrils according to their height in order to further study their morphological properties and persistence length. Persistence length was calculated by fitting the mean‐squared end‐to‐end distance between contour segments. More than 300 fibrillar aggregates were analyzed in the statistical analysis.

### Transmission Electron Microscopy

TEM samples were prepared by placing 5 µL of sample on glow discharged (Emitech K100X, GB) carbon‐coated grids (Quantifoil, D) for 60 sec. After this interval, excess fluid was removed with filter paper and the grid was washed twice. The wet sample was stained in a drop of 2% uranyl acetate for 1 s, followed by a second step of 15 s. Excess moisture was drained with filter paper and the imaging of the air‐dried grids was done in a TEM Morgagni 268 (Thermo Fisher Scientific, USA) operated at 100 kV.

### Scanning Electron Microscopy

Small pieces of the amyloid‐based sample were mounted on SEM aluminum pin stubs with double adhesive carbon tape. The samples were additionally fixed with conductive silver paint (Plano, DE) all around them. After drying, the samples were carefully cut with a razor blade and sputter‐coated with 4 nm of platinum/palladium (CCU‐10, Safematic, CH). SE‐in lens images were recorded at a working distance of 4–5 mm with a SEM (Merlin FE‐SEM, Zeiss, DE), operated at an accelerating voltage of 1.5 kV. For backscatter‐detection, the samples were fixed on double‐adhesive carbon tape and sputter‐coated with 5 nm of carbon (CCU‐10, Safematic, CH).

### Sodium Dodecyl Sulphate‐Polyacrylamide Gel Electrophoresis

The protein samples were dissolved in Milli‐Q water at 4 mg mL^−1^, and then diluted with 2× Laemmli buffer (Bio‐Rad, USA) with the ratio of 1:1 to a final protein concentration of 2 mg mL^−1^. The diluted protein samples were heated at 100 °C for 5 min (non‐reducing condition); mixed with 10% DL‐Dithiothreitol (1 m) to Laemmli buffer and heated at 100 °C for 5 min (reducing condition). Afterward, the protein marker (10 µL) and the protein samples (10 µL) were loaded in each well on the gel (12% separating gel and 5% stacking gel, from Bio‐Rad Laboratories Inc.) at 200 V for 30–40 min. The running buffer for electrophoresis (1 L) contained 3 g Tris, 14.4 g Gly, and 1 g SDS, then the pH was adjusted to 8.3. The gels were stained with Coomassie Blue Fast Staining Solution (Beyotime Institute of Biotechnology Co., Ltd., China). Afterward, the gels were destained using distilled water for 24 h. The sodium dodecyl sulphate‐polyacrylamide gel electrophoresis (SDS‐PAGE) images were analyzed using the gel imaging system (ChemiDoc XRS+, Bio‐Rad, USA).

### Fourier‐Transform Infrared Spectroscopy

The FTIR spectra were obtained by employing a Varian 640 FTIR spectrometer equipped with a Specac Diamond ATR golden gate. Samples were placed on the top of the diamond and were scanned in the range from 4000 to 400 cm^−1^ at a resolution of 1 cm^−1^ at RT. Each spectrum was sampled with 64 scans, and the final analyzed spectrum was averaged with at least three independently acquired spectra.

### Circular Dichroism Spectroscopy

The CD spectroscopy was carried out with a Jasco J‐815 CD spectrometer in a range of 190–280 nm. An aliquot of protein solution (0.02 wt%) was investigated in a high‐quality quartz cuvette with an optical path length of 1 mm in each experiment. An external temperature controller (CDF‐426S) was utilized to stabilize the experimental temperature and to alter the measuring temperature in a range from 20 to 90 °C. The CD spectra were collected at a resolution of 0.2 nm in the continuous scanning mode, and final spectra were obtained by averaging three independently acquired spectra.

### Thioflavin T Assay

A total amount of 100 µL of OG solution with 20 mm ThT was transferred to 96‐well microplate plate (Corning 96‐well plate, half area black/clear bottom polystyrene, nonbinding surface, Corning catalog no.3881, Corning Inc.). The evolution of OG fibrillization in the solution was monitored in a ClarioStar plate reader (BMG Labtech, DE) by recording ThT fluorescence emission at 480 nm with excitation at 440 nm. For measurement of OG fibril formation at 90 °C incubation, the protein solution was heated by means of an oil bath, and an aliquot of sample solution was collected every 20 min and read immediately after collection. For the automatic ThT monitoring test on amyloid fibrillization, ThT signal evolution in protein solution was followed in the plate reader at the maximum temperature (65 °C) or ambient temperature (25 °C) every 20 min or every 2 min.

### Matrix‐Assisted Laser Desorption Ionization Mass Spectroscopy

The samples of mature rigid fibril and worm‐like fibril were purified by MWCO 100 kDa cut‐off. The samples were analyzed by MALDI‐MS. 1 µL of the sample was spotted onto the MALDI target (MTP 384 target polished steel TF, Bruker Daltonics, Bremen, Germany) with 2 µL of *α*‐cyano‐4‐hydroxycinnamic acid (*α*‐CHCA) solution in acetonitrile/water/trifluoroacetic acid (50:50:0.1) for subsequent MALDI‐TOF measurements. The concentration of *α*‐CHCA was 10 mg mL^−1^. MALDI measurements were performed on an ultra fleXtreme MALDI‐TOF/TOF mass spectrometer equipped with a smart beam laser (Bruker Daltonics). The measurement parameters were programmed in flexControl (Version 3.4): laser frequency of 1000 Hz in the positive linear mode with acquisition ranging from 1000 to 70 000 Da. Final spectra consisted of 1000–3000 shots per analysis. The samples were also desalted using C18Zip Tips (Millipore, USA) and analyzed as above.

### Wide Angle X‐Ray Scattering

WAXS experiments were performed using a Rigaku micro‐focused source based on Cu Ka radiation, *λ* = 0.15418 nm. The scattered WAXS intensity was collected by a Fujifilm BAS‐MS 2025 imaging plate system. The sample‐to‐detector distance gives the scattering vector *q* range of ≈0.2–2 Å^−1^ ($q\;=\frac{4\pi }{\lambda }\;\sin\frac{\theta }{2}$, where *θ* is the scattering angle),^[^ ^26^ ^]^ and the corresponding distance $d=\frac{2\pi }{q}$. The in‐plane aligned fibrils were prepared by a casting deposition method to produce a thin membrane of ≈1 mm in thickness. By this approach, amyloid fibrils in the solution were aligned horizontally in the *x–y* plane and randomly located from the top view. The thin‐film samples were placed horizontally in the direction of X‐ray beam in the WAXS measurement.

### Oat Globulin‐Based Aerogel and Hydrogel

OG amyloid fibrils aerogel and hydrogel were fabricated from 2 wt% aqueous amyloid fibril dispersion. The used solutions contained either mature rigid amyloid fibrils obtained from the heating process (90 °C), or the mixture of mature rigid fibrils and worm‐like fibrils yielded from the heating process and a successive incubation at RT (25 °C). Before any further processing, the protein solution was dialyzed for 1 day. For aerogel preparation, an aliquot of amyloid fibril dispersion was freeze‐dried (FreeZone Plus 4.5, Labconco, USA) for 2 days in an aluminum cap. For hydrogel synthesis, the amyloid fibril solution was cross‐linked with a NaCl solution at a final concentration of 450 mm and further dialyzed 2 days at 4 °C.

### Amyloid Fibril Membrane Preparation and Water Purification

To evaluate the ability of oat amyloid fibrils in removing gold and to compare them with *β*‐lg ones, different membranes were prepared by using pure amyloid fibrils and in combination with activated carbon.^[^ ^31b^ ^]^ While the pure amyloid fibrils membranes were formed by vacuum filtration of 1 mL of 0.2 wt% fibril solutions on the surface of cellulose filters, the composite membranes were prepared by vacuum filtration of 1 mL of a homogenous aqueous mixture of 2 wt% amyloid fibril solution (1 mL) and 10 wt% activated carbon solution (2 mL). Then the as‐prepared membranes were used for gold removal from aqueous solutions by bench‐scale dead‐end vacuum filtration setup. In each filtration experiment, 5 mL of aqueous gold solutions were used. Gold concentrations in membrane feed and permeate streams were measured by an AA240Z graphite‐furnace atomic absorption spectroscopy equipped with a GTA 120 graphite tube atomizer and PDS 120 programmable sample dispenser was used. Then, the hybrid membrane efficiency (*E*, %) was calculated by Equation (1):

(1) $$E=\left(1-\frac{C_{p}}{C_{f}}\right)\times 100\%$$

in which *C*_p_ and *C*_f_ stand for gold concentrations in membrane permeate and feed streams (µg L^−1^), respectively.^[^ ^33^ ^]^ Furthermore, to determine the individual role of amyloid fibrils, the adsorption capacity of the pure amyloid fibril's membrane after filtration of gold (2.5 mm) was calculated:

(2) $$q=\frac{\left(C_{p}-C_{f}\right)\times V}{m}$$

where *m* is the mass of amyloid fibrils (g) and *V* is the volume of filtrated gold solution (L).^[^ ^34^ ^]^

### Amyloid‐Based Electrodes

Freshly prepared OG amyloid solution with mature rigid fibrils was first diluted to a concentration of 0.2 wt% and mixed with 25‐nm Au NPs (5 mg mL^−1^, Sigma). Then, the amyloid fibril/Au NPs solution was enhanced by mixing with an HAuCl_4_ solution, followed by adding a reducing agent (hydroxylamine hydrochloride)^[^ ^29a^ ^]^ droplet‐by‐droplet and waiting for 15 min at ambient condition. The final solution contained 0.1 wt% OG amyloid fibrils, 2 µg mL^−1^ Au NPs, unreacted HAuCl_4_, and hydroxylamine at the concentration of 50 and 100 mg mL^−1^, respectively. Then, the solution was centrifuged (8000 rpm) for 10 min and the precipitate was collected and transferred to a homemade electrode template made by water‐proof tape with an electrode shape. The patterned interdigital electrode was dried by nitrogen flow and then further dried in a desiccator. The capacitance of the interdigital electrode was measured with a high‐precision LCR meter (Hioki‐3536, HIOKI, Japan) at a voltage of 1 V and a frequency of 100 kHz at RT in ambient conditions.

## Conflict of Interest

The authors declare no conflict of interest.

## Author Contributions

J.Z., T.L., L.W., and R.M. conceived the idea; J.Z. and R.M designed the experiments; J.Z., T.L., M.P., V.L.‐B., and J.T. performed the experiments and collected the data; J.Z. and M.U. analyzed the data; R.M. designed and directed the study. J.Z., M.U., M.P., and R.M. wrote the manuscript, with contributions from all the other authors.

## Supporting information

Supporting Information

## Data Availability

The data that support the findings of this study are available on request from the corresponding author.
