# Supplementary material for: Oat Plant Amyloids for Sustainable Functional Materials
Source: Adv Sci (Weinh). 2021 Dec 20;9(4):2104445. doi: 10.1002/advs.202104445 (PMC8811842; doi:10.1002/advs.202104445)
Supplement: Supplementary file 1 — Supporting Information [file ADVS-9-2104445-s001.pdf]

ADVANCED SCIENCE
Open Access

Supporting Information

for *Adv. Sci.*, DOI: 10.1002/advs.202104445

Oat plant amyloids for sustainable functional materials

*Jiangtao Zhou, Ting Li, Mohammad Peydayesh, Mattia Usuelli, Viviane Lutz-Bueno, Jie Teng, Li Wang, Raffaele Mezzenga**

Supporting Information

Oat plant amyloids for sustainable functional materials

*Jiangtao Zhou, Ting Li, Mohammad Peydayesh, Mattia Usuelli, Viviane Lutz-Bueno, Jie Teng, Li Wang, Raffaele Mezzenga**

Figure S1. Description of oat globulin structure and protein sequence

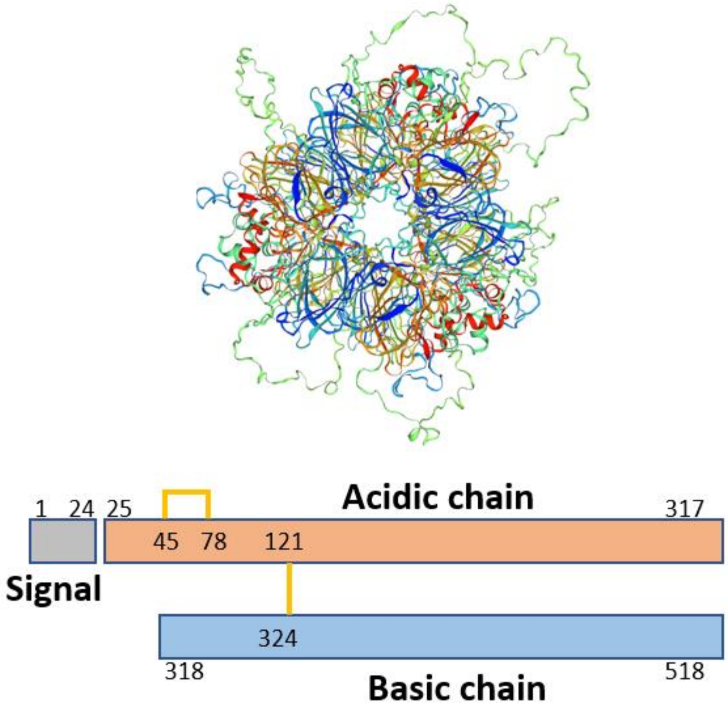

Figure S1. Oat-globulin structure and its protein sequence. Oat globulin, the primary storage oat protein, normally exists in the oat grain as a 329 kDa hexamer that consists of 54 kDa monomeric globulin[1] linked by noncovalent interactions.[2] The crystalline structure of oat globulin has not been reported, and this schematic representation (upper) shows the structure of oat globulin hexamer from

(*Avena sativa*) 12S seed storage globulin structure (P12615, Swiss-Model), templated from its homology model, coconut allergen cocosin (5wpw.1.E Swiss-Model). Protein sequence information (lower) of oat globulin indicates monomeric oat globulin contains a signal peptide of 24 amino acids, followed by an acidic polypeptide of 293 amino acids and a basic polypeptide of 201 amino acids,[3] with molecular weight of acidic and basic chains of 32 and 22 kDa respectively. These two chains are connected by an interchain disulfide bond, and another disulfide bond is present in the acidic chain.[4]

Figure S2. The diversity of proteins from different plant sources

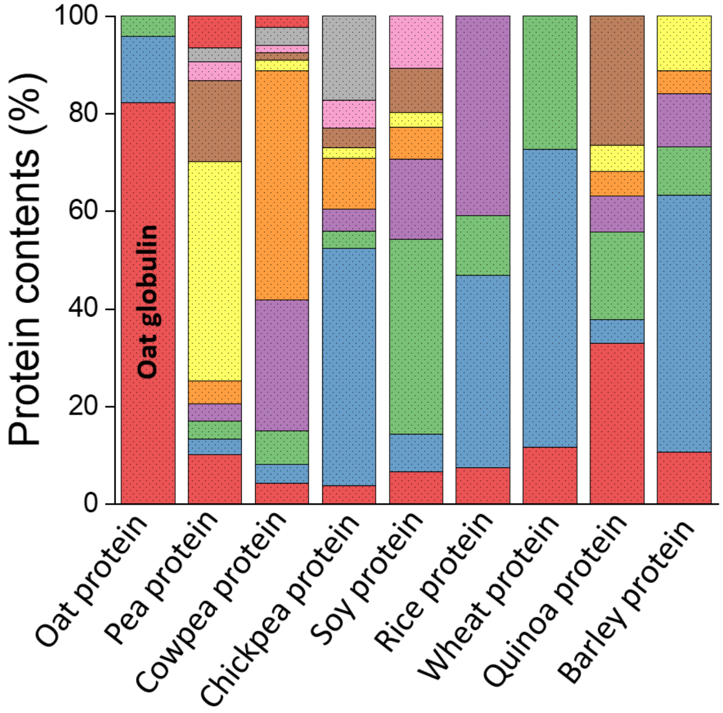

Figure S2. The diversity of protein content from different plant sources. The protein from oat, pea, cowpea, chickpea, soy, rice, wheat, quinoa and barley plants were extracted and analysed through a SDS-PAGE test (Figure 1d). Based on the patterns extracted from the SDS page, protein fractions from different sources were analyzed. As seen, oat globulin accounts for up to 80% in the oat protein, while proteins from other sources show a large diversity in their protein composition.

Figure S3. AFM and TEM characterization of oat-globulin mature rigid fibrils

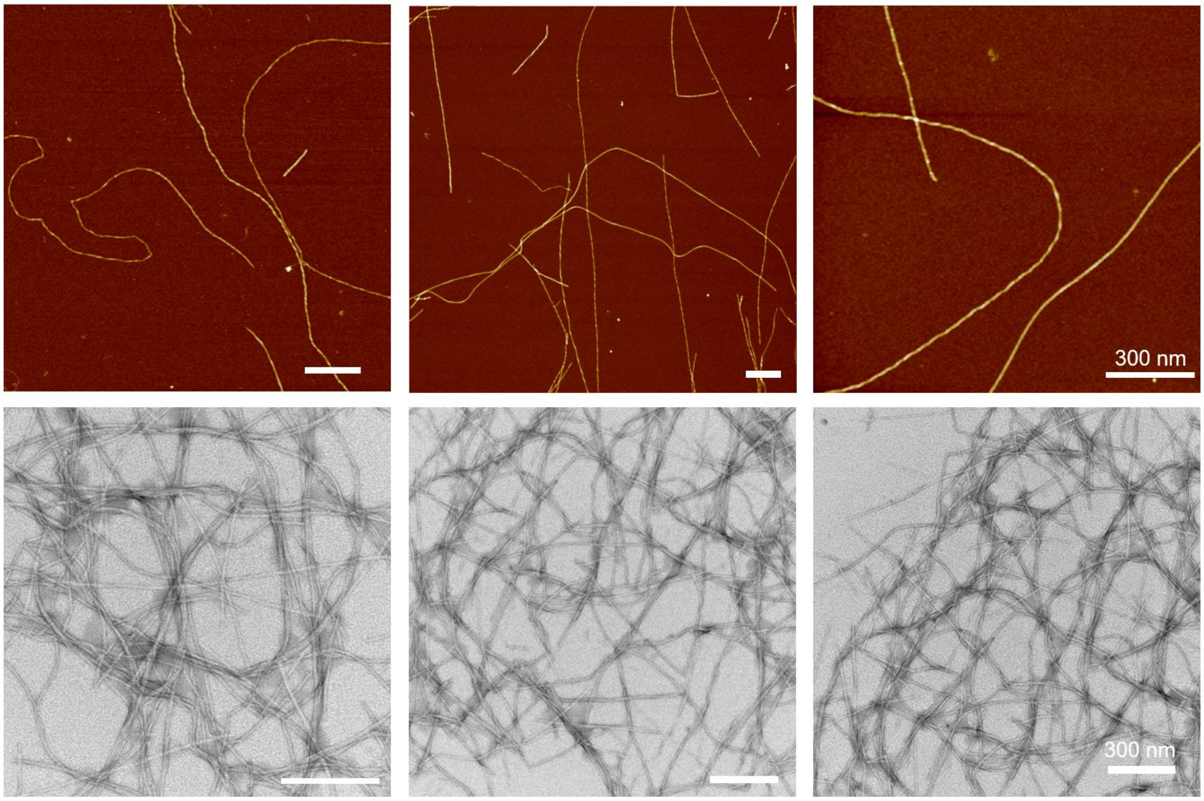

Figure S3. AFM and TEM characterization of oat globulin mature rigid fibrils. These fibrils are obtained by heat-denaturation at 90 °C and immediately diluted to the concentration of 0.02wt% before imaging. The AFM (up) and TEM (bottom) images indicate that these mature fibrils show a fluctuating height profile and could reach several micrometers in length.

Figure S4. Zeta-potential of oat globulin

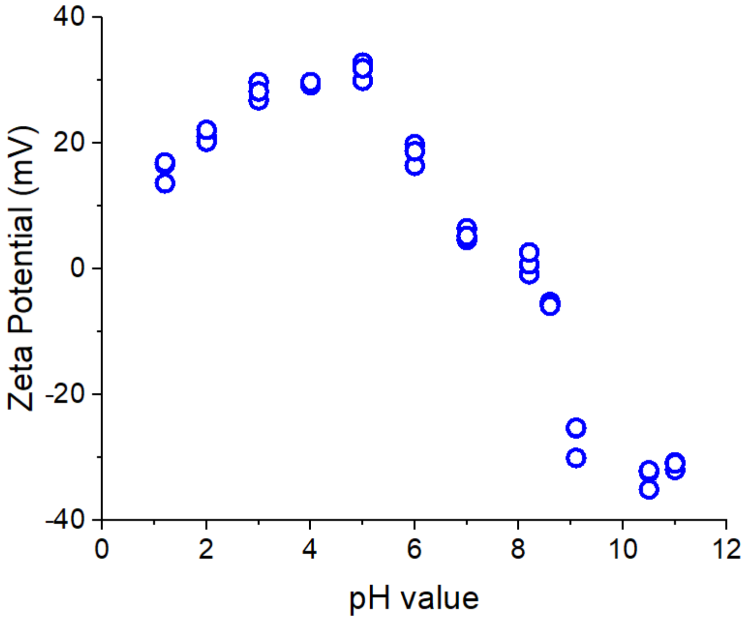

Figure S4. Zeta-potential of oat globulin. The plot shows that the isoelectric point of plant-derived oat globulin is around pH 7, and that the oat globulin is positively charged with a value around 20 mV under the incubation condition at pH 2. The value might be slightly lower than that it should be at low pH (pH<3), and this is due to the reaction of copper electrodes with hydrogen ions in the solution during measurement.

Figure S5. Polymorphisms of oat-globulin amyloid fibrils

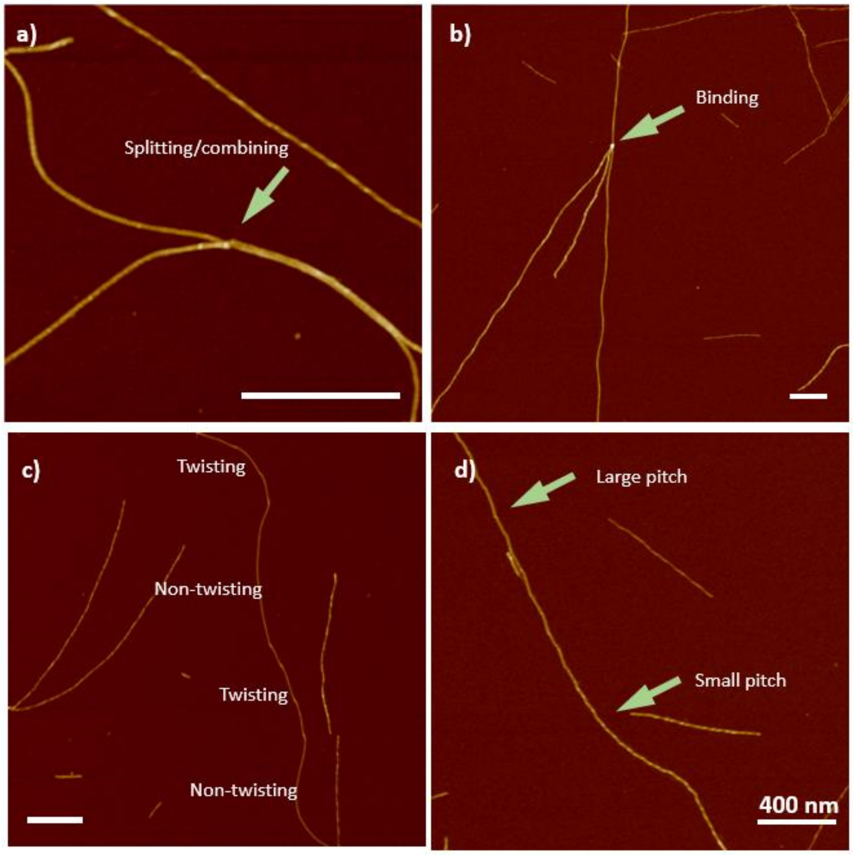

Figure S5. Amyloid polymorphism of oat-globulin self-assembly. (a-b) Illustration of fibril-fibril interaction that shows side-by-side packed ribbon-like fibrils in either a splitting or combining process (a), and multiple fibril binding at the same location (b). (c-d) Pitch variation within self-twisting ribbon like amyloid fibrils. This phenomenon is believed to happen during the process of fibril formation, when the dynamic balance between intrinsic chirality of protein building blocks and fibril mechanical strain to suppresses twisting has not reach the equilibrium.

Figure S6. Snapshots the inter-fibril interaction among mature fibrils

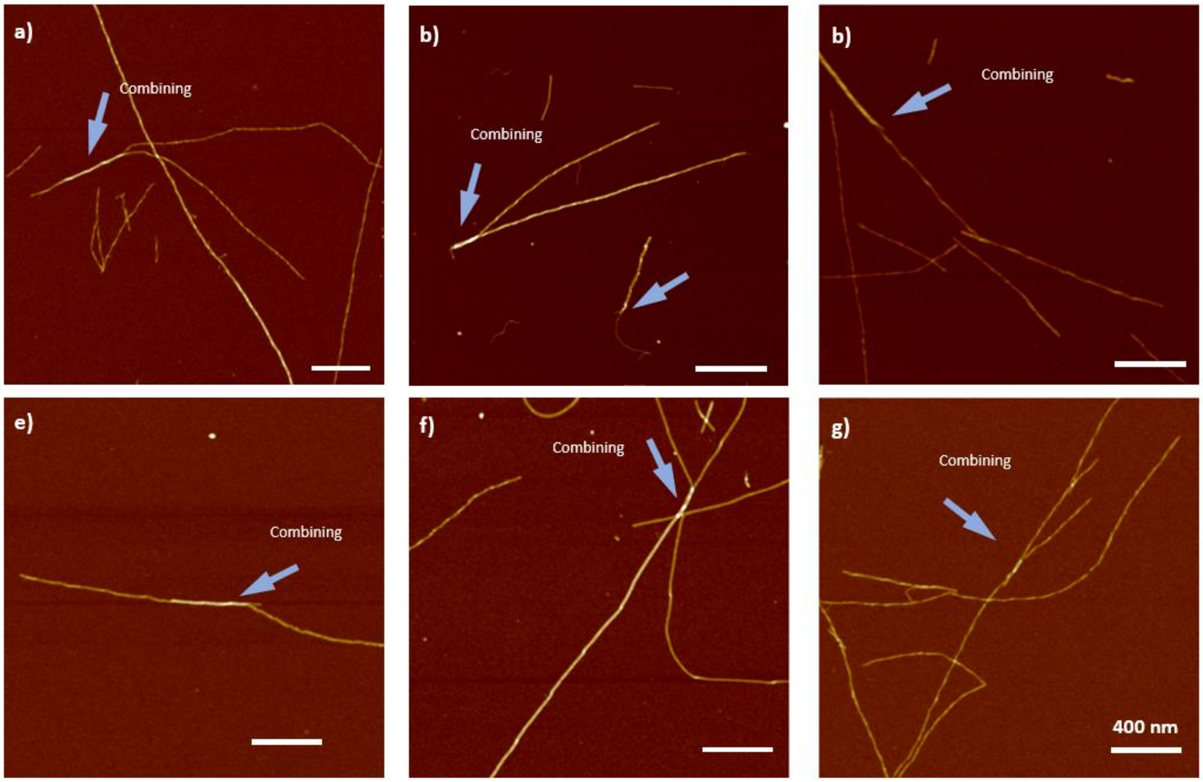

Figure S6. AFM images unravel the fibril-fibril events during the fibrillization process. (a-f) The twisting ribbon-like fibrils turn into thicker rigid fibrils at the region indicated by the arrow, as a consequence of combination of mature rigid fibrils. The combined fibrils in the AFM images also exhibit a clear fluctuating height profile, that suggests their self-twisting scheme.

Figure S7. Persistence length of each family of mature fibrils

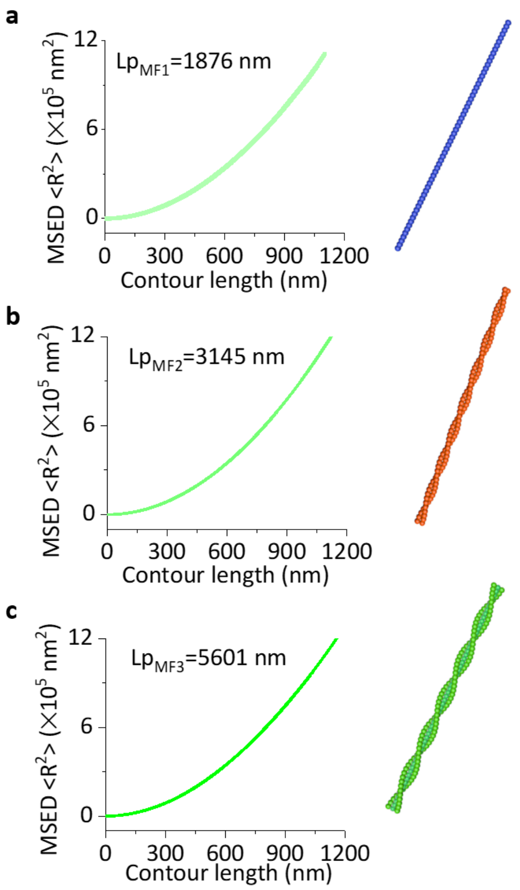

Figure S7. The persistence length of mature oat-globulin amyloid fibrils. The persistence length was computed from the mean-squared end-to-end distance (MSED) between contour segments, according to a theoretical dependence: $< R^2 >= 4\lambda[l - 2\lambda(1 - e^{-l/2\lambda})]$,[5] where λ is the persistence length and R is the direct distance between any pair of segments along a contour separated by an arc length l. (a-c) The average persistence length of mature oat globulin fibrils that belong to different families is in the order of several micrometers: the values extracted from fitting are 1.8, 3.1 and 5.6 µm for single-, double- and triple-stranded fibril families, respectively.

Figure S8. Oat-globulin worm-like fibrils and their persistence length

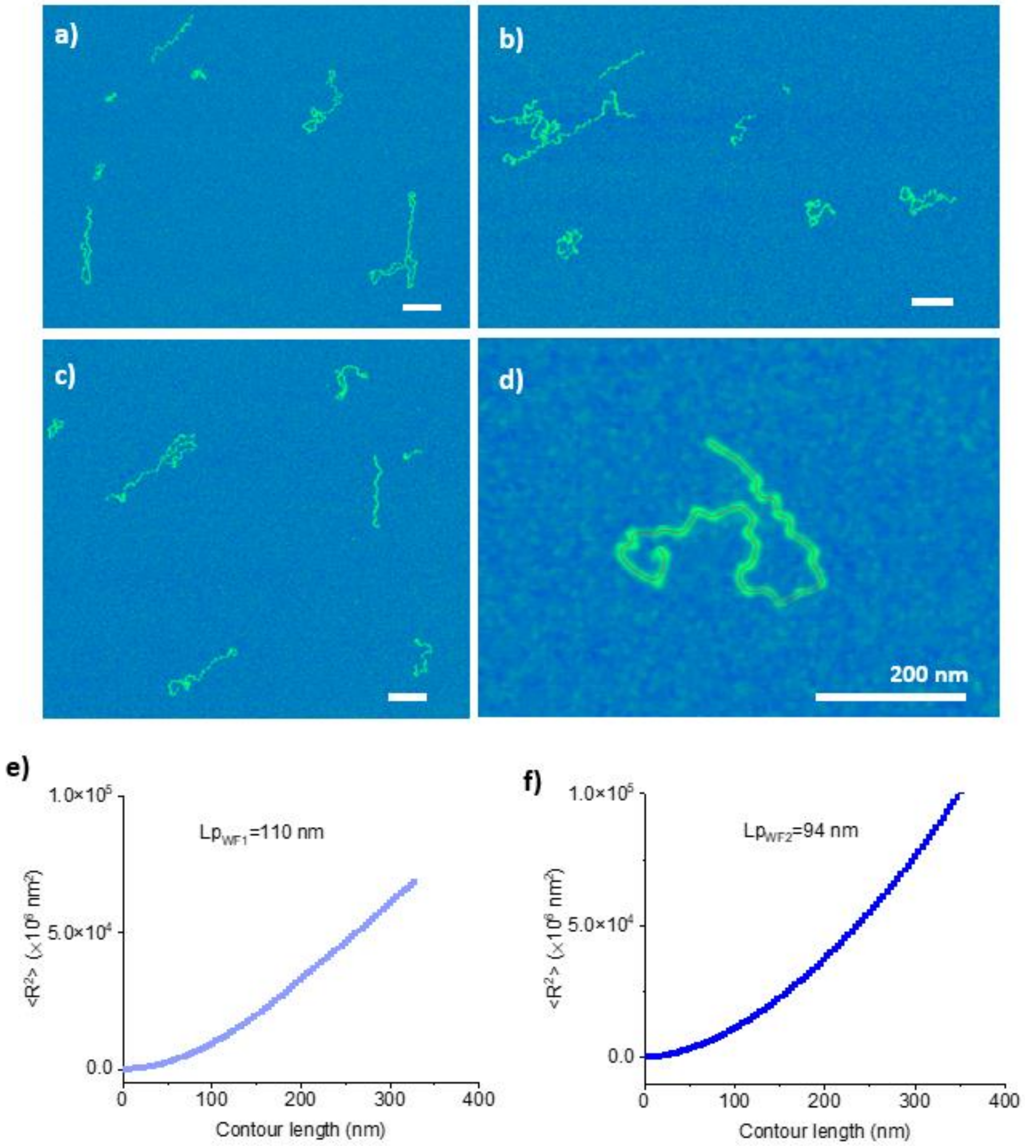

Figure S8. Oat-globulin worm-like fibrils and their persistence length. (a-d) AFM images of long worm-like fibrils incubated after 50 days. These fibrils showed a highly flexible shape, and a contour length up to more than 500 nm. The zoomed-in image (d) indicates a worm-like fibril with extracted height profile (black line) along the ridge of fibril traced by FiberApp.[5] (e, f) Persistence length of both families (WF1 and WF2) of worm-like fibrils, calculated by fitting the mean-squared end-to-end distance (MSED) between contour segments. The persistence length of single- and double-stranded worm-like fibrils is around 100 nm, one order of magnitude lower than that of mature fibrils. This bond-correlation analysis suggests a much weaker and flexible amyloid core in worm-like fibrils.

Figure S9. FTIR spectra of mature rigid fibrils, worm-like fibrils and their hybrid systems

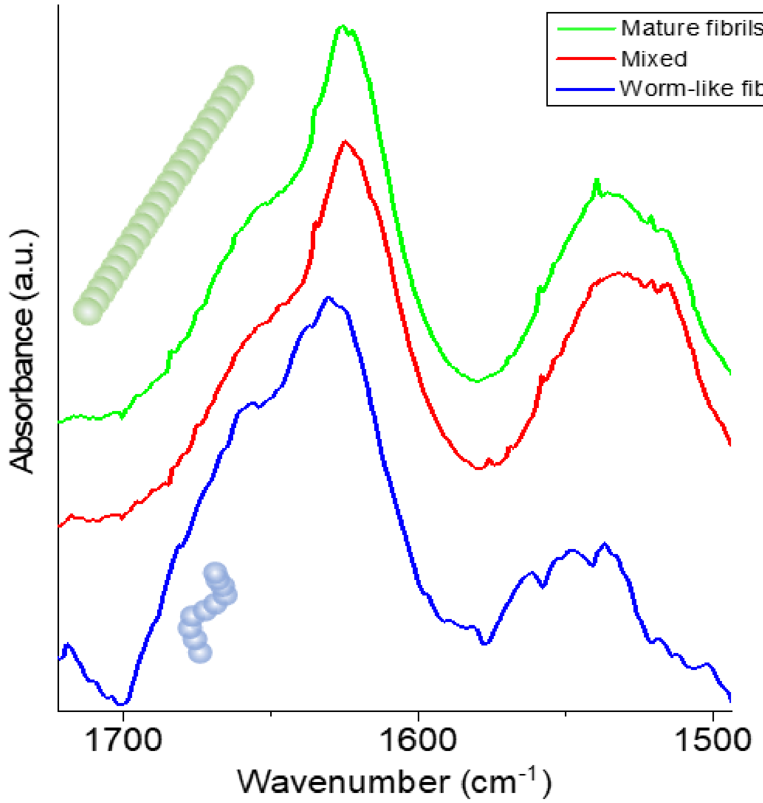

Figure S9. FTIR spectra in the protein amide I and amide II wavenumber regions of oat-globulin mature rigid fibril, worm-like fibril and their hybrid systems. As seen, these fibrils show a dominating conformation of β-sheet that is peaked at 1620-1630 cm^{-1}, as a consequence of the characteristic β-sheet secondary structure that composes the core of amyloid fibrils. In addition, a shoulder at 1640-1660 cm^{-1} can be found in each spectrum and is related to random coil and α-helix conformations. In the case of worm-like fibrils, this shoulder extends over a wider wavenumber range. This is due to the lower concentration of worm-like fibril, and thus a relatively lower ratio between worm-like fibril and surrounding polypeptides with random coil and α-helix, compared to the other two cases.

Figure S10. CD spectra of dialyzed mature rigid fibril, worm-like fibril and their hybrid systems

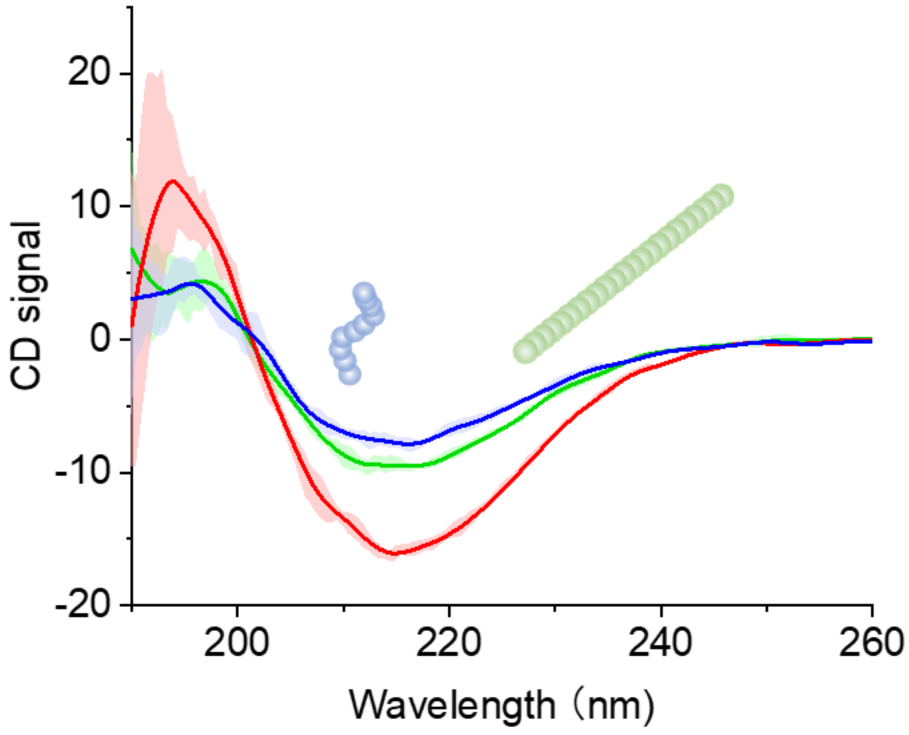

Figure S10. CD spectra of dialyzed mature rigid fibril (green), worm-like fibril (blue) and their hybrids systems (red). The primary fingerprint on the spectra indicates that the mature fibril, worm-like fibril and their hybrids contain a rich β-sheet structure featured by the charactering absorption dip at 218 nm. The errors from triplicates are indicated with the spectra.

Figure S11. Estimation of the yield of oat globulin amyloid.

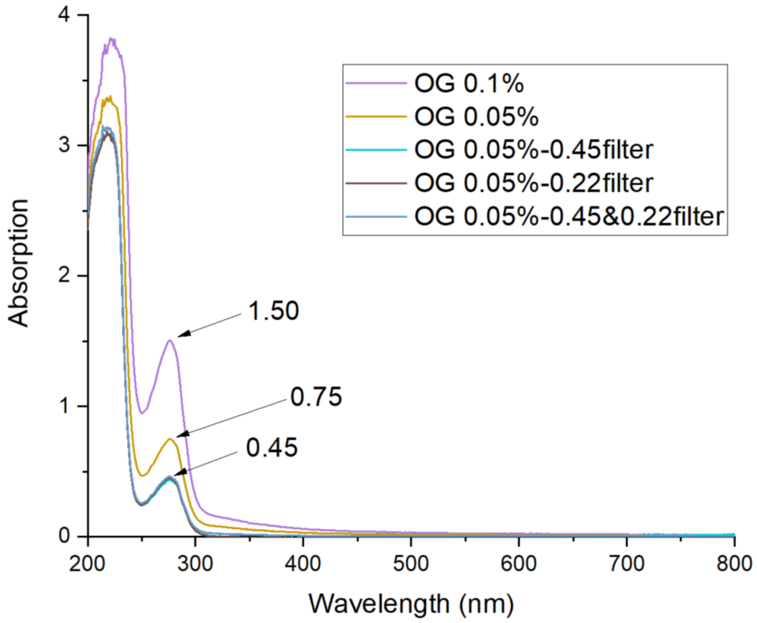

Figure S11. Estimation of the yield of oat globulin amyloid. The yield of oat globulin amyloid fibril from native oat globulin protein solution was estimated by using UV-visible spectroscopy. The OG amyloid solution (0.05wt%) and three filtered 0.05wt% OG amyloid solution (by a filter of either 0.45 µm, or 0.22 µm, or 0.45 µm pore size followed by another filtration at 0.22 µm pore size) were tested. Taking the absorption peak at 280nm of amyloid solution (a) and the filtrate of amyloid solution (b) as *Abs(a)* and *Abs(b)*, respectively, the estimated yield of oat globulin amyloid was estimated as: $\frac{Abs\ (a) - Abs\ (b)}{Abs\ (a)} \times 100\% = \frac{0.75-0.45}{0.75} \times 100\% = 40\%$. To prove the peak at 280nm in linear absorption region, we measured double-concentrated amyloid solution (0.1wt%) and found the absorption also doubled (1.50) at 280nm.

Figure S12. Oat globulin amyloid based hydrogel

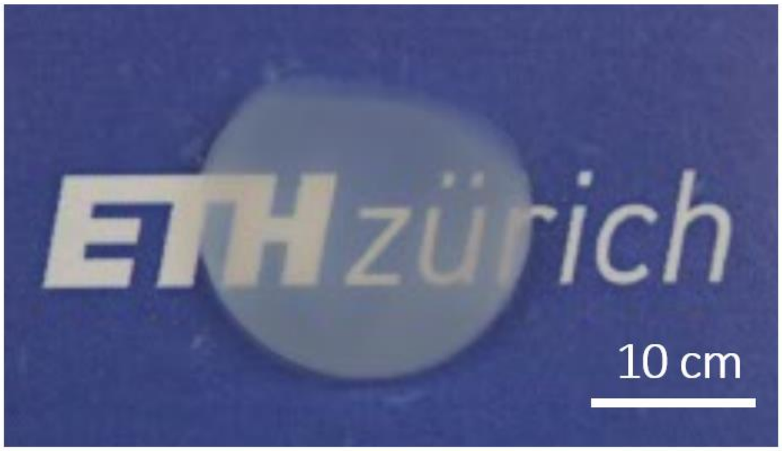

Figure S12. Hydrogel synthetized from oat globulin amyloid fibrils. The semi-transparent hydrogel was cross-linked by mixing the amyloid fibril solution with an NaCl solution at a final concentration of 450 mM and was dialyzed though a semi-permeable membrane for 2 days.

Figure S13. Comparison of heavy metal adsorption capacity between β-lactoglobulin fibrils and oat-globulin fibrils

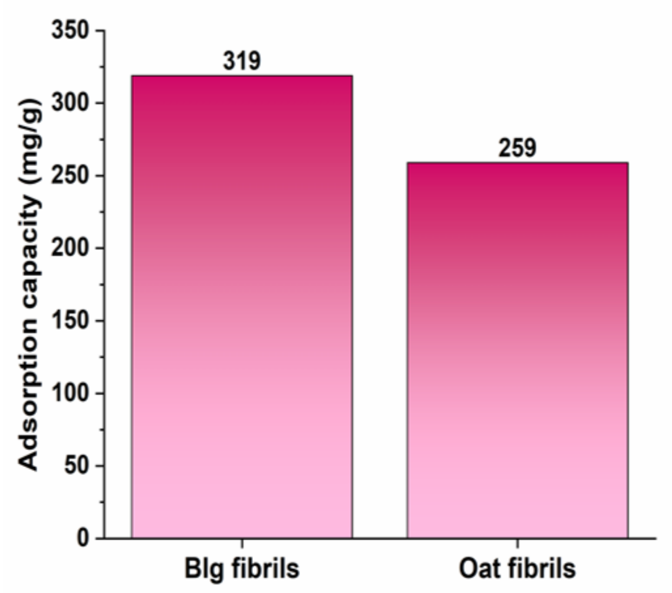

Figure S13. Adsorption capacity of heavy metal of β-lactoglobulin and oat-globulin amyloid fibrils. The animal-based β-lactoglobulin is commonly used to form amyloid fibrils with high-performances in the water purification and heavy metal removal technology.[6] In comparison, a slightly lower but comparable performance in the heavy metal removal can be seen in the case of oat globulin fibrils.

Figure S14. Pressure-sensitive AuNP-amyloid hybrid electrode

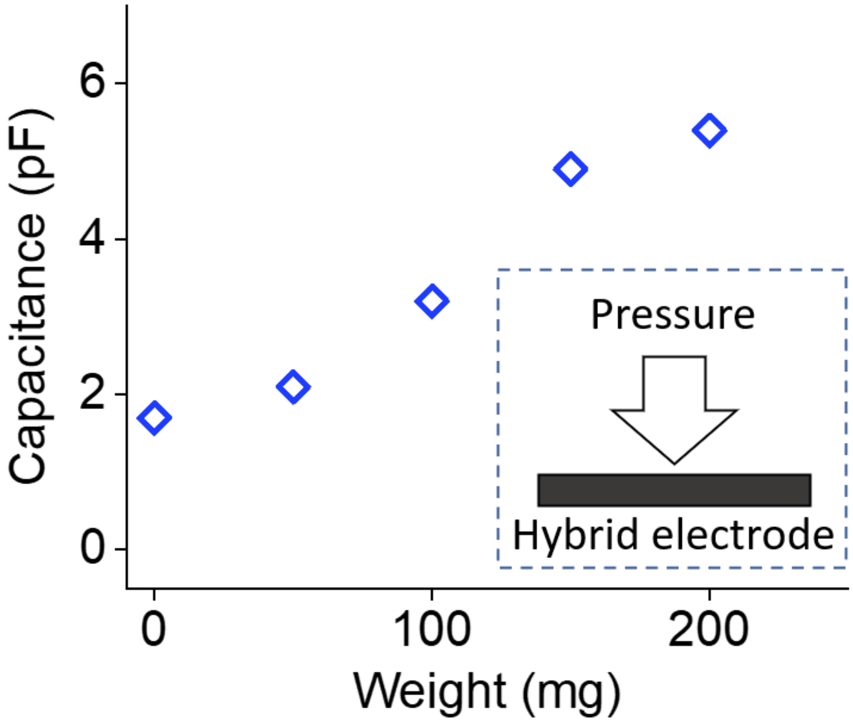

Figure S14. Pressure sensitive AuNP-amyloid hybrid electrode. Oat-globulin amyloid fibrils were coated with gold-NPs and further treated with a gold enhancement process, before patterning into the interdigital electrode. We detected an increase of electrical capacitance of this electrode with a larger weight applied on it. This sensitivity is believed to be attributed to the soft and porous structure of the AuNP-amyloid hybrid material.

Figure S15. Electrical conductivity of AuNP-amyloid material

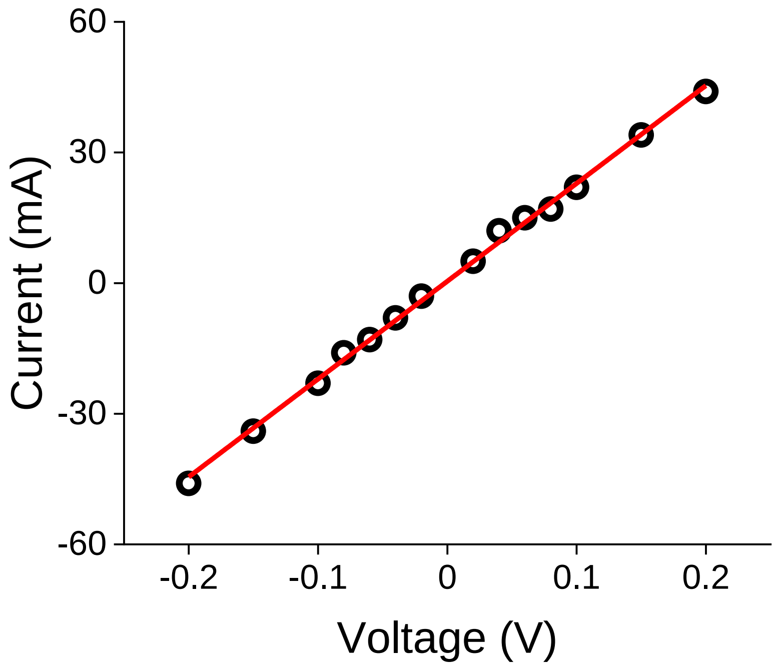

Figure S15. Electrical conductivity of AuNP-amyloid material. We measured the electrical property of a conductive wire made of this AuNP-amyloid hybrid material with a length of 10 mm. The measured results indicate a relatively high electrical conductivity and outline the possibility of applying this AuNP-amyloid material in bioelectric devices.

References of Supporting Information:

[1] O. E. Mäkinen, N. Sozer, D. Ercili-Cura, K. Poutanen, *Sustainable Protein Sources* (Eds.: S.R. Nadathur, J.P.D. Wanasundara, L. Scanlin), Academic Press, San Diego, **2017**, pp. 105–119.

[2] a) R. Casey, in *Seed Proteins* (Eds.: P.R. Shewry, R. Casey), Springer Netherlands, Dordrecht, **1999**, pp. 159–169; b) M. A. Shotwell, *Seed Proteins* (Eds.: P.R. Shewry, R. Casey), Springer Netherlands, Dordrecht, **1999**, pp. 389–400.

[3] M. A. Shotwell, C. Afonso, E. Davies, R. S. Chesnut, B. A. Larkins, *Plant Physiol.* **1988**, *87*, 698–704.

[4] S. R. Burgess, P. R. Shewry, G. J. Matlashewski, I. Altosaar, B. J. Miflin, *Journal of Experimental Botany* **1983**, *34*, 1320–1332.

[5] I. Usov, R. Mezzenga, *Macromolecules* **2015**, *48*, 1269–1280.

[6] a) S. Bolisetty, M. Peydayesh, R. Mezzenga, *Chem. Soc. Rev.* **2019**, *48*, 463–487; b) S. Bolisetty, R. Mezzenga, *Nature Nanotech* **2016**, *11*, 365–371.
